# Clinicopathologic correlation and interdependence of basic patterns of placental injury

**DOI:** 10.1007/s00428-025-04073-x

**Published:** 2025-03-29

**Authors:** Jerzy Stanek, Dustin Funk

**Affiliations:** Division of Pathology, Cincinnati Children’s Medical Center, 3333 Burnet Avenue, Cincinnati, OH 45229-3025 USA

**Keywords:** Placenta, High-risk pregnancy, Maternal vascular malperfusion, Fetal vascular malperfusion, Inflammation

## Abstract

Placental lesions rarely occur in isolation and placental lesion multiplicity is associated with poorer pregnancy outcome than that of isolated lesions. As little is known about mutual relations of various patterns of placental injury simultaneously occurring in the same placentas, particularly in relation to gestational age, this retrospective observational analysis was undertaken to study those in a population of 2486 cases of the second half high-risk pregnancy dominated by fetal congenital anomalies. To this end, 23 independent clinical and 48 placental phenotypes were statistically compared among 6 basic patterns of placental injury: Group 1: acute inflammation, Group 2: chronic inflammation, Group 3: maternal vascular malperfusion, Group 4: fetal vascular malperfusion, large vessel, Group 5: fetal vascular malperfusion, distal villous, and Group 6: shallow placental implantation. All cases had E cadherin/CD34 immunostaining performed for the diagnosis of recent fetal vascular malperfusion. There was a significant overlap among the studied patterns and lesions of placental injury. Placental distal villous fetal vascular malperfusion and acute inflammation was most frequently statistically significantly associated with abnormal clinical conditions, while lesions of distal villous fetal vascular malperfusion and maternal vascular malperfusion with other placental lesions/patterns of injury. The double immunostaining was responsible for the fetal vascular malperfusion being the most common type of placental injury in this population of placentas. The acute inflammation best correlated with clinical condition in preterm pregnancy and distal villous fetal vascular malperfusion at term. Maternal vascular malperfusion plus the above two patterns of placental injury correlated best with other placental phenotypes in mid third trimester.

## Introduction

Placental examination is regarded as a basic tool in evaluation of the pregnancy outcome and fetal/neonatal condition [1–5]. Histological examination of the placenta, especially for malperfusion disorders, is crucial in elucidating pathways to fetal and neonatal deaths in preterm infants [5]. The clinical-pathology correlation must be understood to communicate the conclusions to the patient. In the last years, placental lesion terminology has been adopted at the Amsterdam conference [6]. Overall, 4 major patterns of placental injury were distinguished (acute inflammatory, chronic inflammatory, maternal vascular malperfusion and fetal vascular malperfusion) [7]. To those, shallow placental implantation was recently added by the author [8].

The normal placental histomorphology and the frequencies of various abnormal histology patterns vary with gestational age (GA) [9], e.g. villitis of unknown etiology (VUE) and fetal vascular malperfusion (FVM) are most common in term and late preterm pregnancies, maternal vascular malperfusion (MVM) in early and late preterm pregnancies, acute chorioamnionitis in early preterm previable pregnancies [7, 10–12], and shallow placental implantation (SPI) peaks in mid-third trimester [8]. Overall, placental clinicopathological associations are strongest for the second trimester. As a consequence, placental histology normal in preterm pregnancy may be abnormal at term and vice versa. In addition, various patterns of placental injury can and do coexist in same placenta (overlap patterns) [13], and placental lesion multiplicity is more likely to result in complicated pregnancy outcome and fetal/neonatal compromise that a single abnormal pattern/lesion [14]. Furthermore, certain clinical conditions and placental lesions tend to recur in subsequent pregnancies [13]. The awareness of gestational age dependance of some clinical conditions and placental lesions and patterns of injury may be useful in a meaningful clinicopathological explanation of perinatal outcome in individual cases by comparing the observed clinical and placental phenotypes with the dendrograms of multi-dimensional scaling [15].

However, little is known about mutual relations of various patterns of placental injury, in relation to gestational age, the analysis of which is the topic of this study.

## Methods

**Fig. 1:**
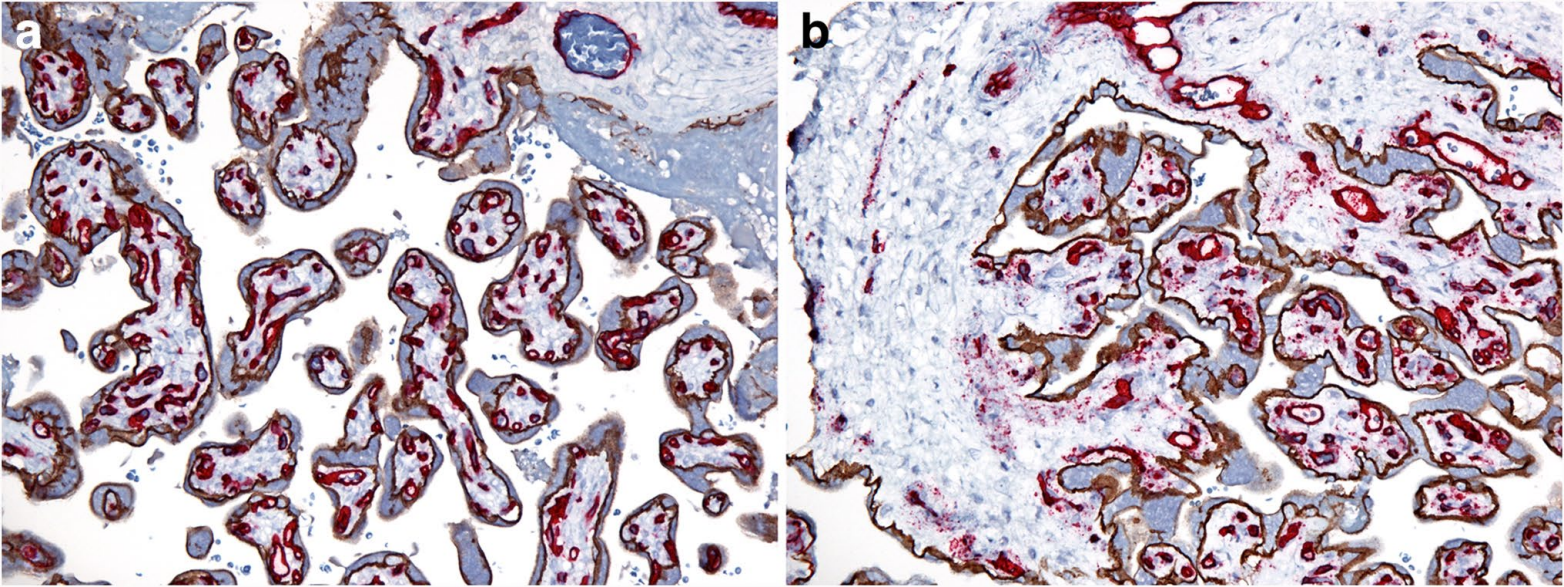
Normal and abnormal pattern of E-cadherin/CD34 immunostaining (E-cadherin brown, CD34 red). Objective magnifications × 20, term pregnancies, grossly and microscopically (hematoxylin–eosin) normal placentas, (**a**) gastroschisis, normal distal villi, (**b**) congenital diaphragmatic hernia, distal villi with endothelial fragmentation (red haziness in villous stroma by CD34) diagnostic of recent distal fetal vascular malperfusion (about two days duration before delivery)

The placentas were submitted for examination by clinicians, based on the high-risk nature of pregnancy and/or abnormal gross placental morphology. Sampling, gross and microscopic criteria of placental examination recommended by the Amsterdam consensus conference [6] were generally applied, with the additions introduced by the author [8, 16–18]. In particular, the recent fetal FVM lesions were those with clustered distal villous endothelial fragmentation by CD34 immunostaining [19, 20] (Fig. 1). The established FVM was diagnosed when clustered avascular/hyalinized/sclerotic villi were present on hematoxylin–eosin (H&E)-stained slides [6], and the remote FVM when clusters of mineralized distal villi were seen on H&E-stained slides or iron/von-Kossa staining [21]. The ongoing distal villous FVM was diagnosed when one, two or all the above three distal villous FVM patterns were present is the same placenta, adjacent to one another, separately from each other on the same slide, or different histology slides from the same placenta [22].

**Fig. 2:**
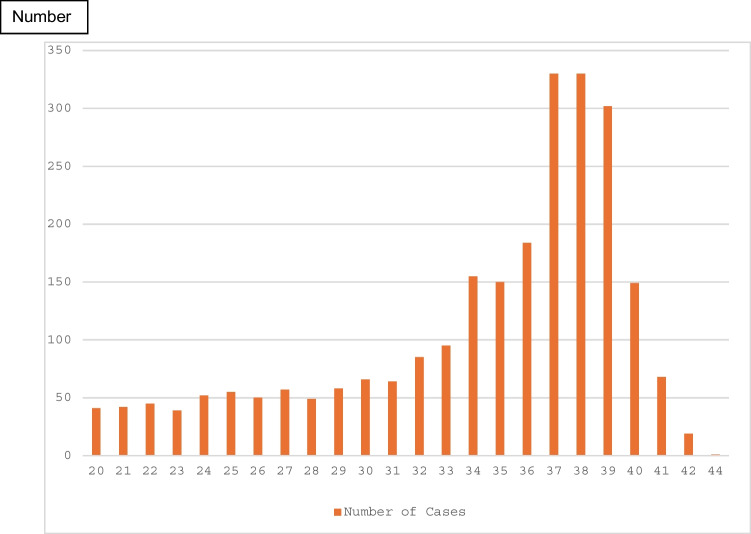
Distribution of frequencies of cases vs gestational. age at delivery (weeks)

Placental reports of all 2486 consecutive pregnancies ≥ 20 weeks GA in which double immunostaining was performed on at least one grossly unremarkable placental section of placentas received since 2006, were reviewed. The latter inclusion criterion was to ensure the uniformity and comparability of the conditions analyzed. The diagnoses studied are those contained in the original final placental reports which were not changed for the purpose of this study. Twenty-three independent clinical and 48 placental phenotypes (variables, patterns, lesions) were statistically analyzed with the ANOVA or Chi-square, where appropriate, with application of the Bonferroni correction for multiple comparisons, p Bonferroni < 0.002 being adopted as the threshold of statistical significance. Cases with GA below 20 weeks were excluded because of low case numbers of cases and unknown significance of double immunostaining results at that GA. As in previous author’s publications, stillbirths were not excluded. Figure 2 shows the GA distribution of all cases studied. Most pregnancies ended in term or late pre-term period.

Table 1 shows the frequencies of the 6 analyzed patterns of placental injury and average frequencies of the individual components of the patterns: 4 basic patterns [7], with the FVM subdivided into the large vessel and distal villous types because of different etiopathogenesis, timing and prognosis [6, 23, 24], plus SPI [8] were compared. Also, other placental lesions not being components of the above-mentioned 6 groups were statistically compared separately.

**Table 1:** Basic patterns and lesions of placental injury

Variable	Nr. of placentas
Acute chorioamnionitis	781
Acute subchorionitis	334
Maternal inflammatory reaction	204
Fetal inflammatory reaction	243
Chronic inflammation	412
Villitis of unknown etiology	334
Plasma cell deciduitis	121
Maternal vascular malperfusion	1005
Hypertrophic decidual arteriopathy	519
Hyaline necrosis/atherosis	133
Infarction, established (> 5% of placental volume)	257
Intravillous hemorrhage	112
Chronic hypoxic injury (pre-uterine, uterine, post-tuterine)	477
Proximal (large vessel) fetal vascular malperfusion	1402
Fetal vascular ectasia	1076
Luminal vascular thrombi (occluding or non-occluding)	620
Intramural fibrin deposition	334
Stem vessel obliteration	511
Distal villous fetal vascular malperfusion	796
Clustered endothelial fragmentation/stromal vascular karyorrhexis (recent FVM)	322
Clustered avascular/sclerotic villi (established FVM)	616
Clustered villous mineralization (remote FVM)	160
Shallow placental implantation	1300
Membrane chorionic microcysts	350
Increased extravillous trophoblasts in chorionic disc	484
Chorionic disc chorionic microcysts	441
Placenta creta (occult, basal plate myometrial fibers)	305
Decidual multinucleate trophoblastic giant cells, clustered	565

## Results

The mean GA was 34 weeks (mid-preterm range). Covariance of individual lesion numbers within the 6 patterns of placental injury listed in Table 1 are showed in Table 2: acute inflammation (3 lesions: acute subchorionitis; acute chorioamnionitis, maternal inflammatory reaction; acute chorioamnionitis, fetal inflammatory reaction); chronic inflammation (2 lesions: chronic villitis of unknown etiology, chronic plasma cell deciduitis); MVM (5 lesions: hypertrophic decidual arteriopathy, hyaline necrosis/atherosis of spiral arteries; established villous infarction, intra-villous hemorrhage, and a chronic hypoxic pattern of placental injury); FVM, large proximal vessel (4 lesions: fetal vascular ectasia, fetal vascular thrombi, intramural fibrin deposition, stem vessel obliteration); distal villous FVM (3 lesions: clustered endothelial fragmentation by CD 34 immunostaining (Fig. 1), avascular villi, and clustered villous mineralization); and SPI (5 lesions: membrane chorionic microcysts, increased cell islands, chorionic disc microcysts, clustered decidual of multinucleate trophoblasts, and sub-clinical placenta creta, i.e. occult placenta accreta and basal plate myometrial fibers. The averages of numbers of lesions of all variables were in the range of 1–2 in each group. The kurtosis (a measure of flatness of distribution) and the skewness (a measure of asymmetry of distribution) were high for chronic inflammation and MVM and significantly lower for other variables.

**Table 2:** Covariance of gestational age and average numbers of  individual placental lesions in 6 analyzed patterns of placental injury

Variable	Gestational age (weeks) ± SD	Acute chorioamnionitis: 3 lesions	Chronic inflammation: 2 lesions	Maternal vascular malperfusion: 5 lesions	Fetal vascular malperfusion, proximal: 4 lesions	Fetal vascular malperfusion, distal: 3 lesions	Shallow placental malperfusion: 5 lesions
Average ± SD	34.3 ± 5.4	1.9 ± 0.9	1.1 ± 0.06	1.5 ± 0.8	1.8 ± 0.5	1.4 ± 0.6	1.6 ± 0.9
Kurtosis	0.10	− 1.6	4.8	2.8	0.0	0.5	0.9
Skeweness	− 1.0	0.2	2.6	1.8	0.9	1.2	1.2

**Fig. 3:**
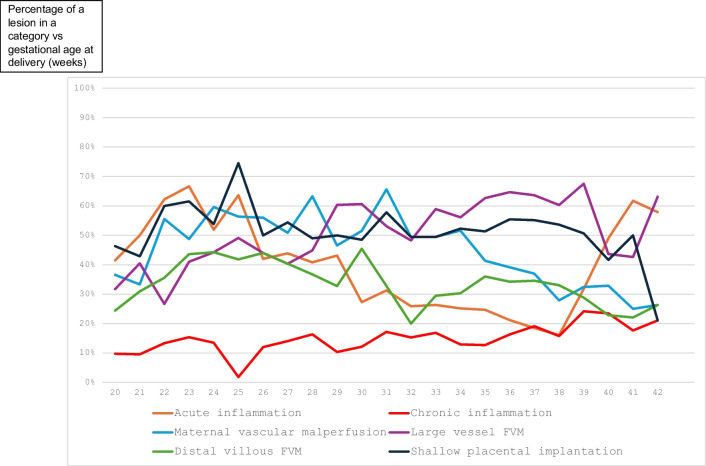
Distribution of lesions of 6 patterns of placental injury (at least one lesion from a group) according to gestational age at delivery (weeks)

**Fig. 4:**
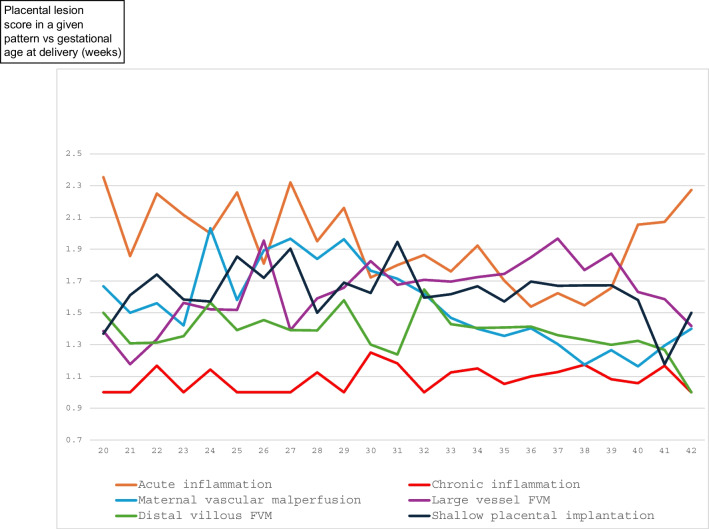
Gestational age at delivery (weeks) distribution of average numbers of lesions (listed in Table 1) per category of patterns of placental injury

Figure 3 shows the GA distribution of the 6 analyzed patterns (at least one lesion from each group). The distribution was bimodal for acute inflammation and proximal FVM (peaking in second trimester and term pregnancies). Only the lesions of SPI, distal FVM and MVM were most common in preterm third trimester (the latter corresponding to occurrence of hypertensive conditions) and decreasing in frequency at term. Chronic inflammatory lesions were most common at term. In comparison, Fig. 4 shows the GA distribution of the intensity (scores) of the 6 patterns of placental injury analyzed in Table 2. The most striking observation is that acute inflammation is equally common in early preterm and in term pregnancies, but the latter is only mild (acute subchorionitis) with low scores.

The analysis of clinical phenotypes/variables among the 6 groups is presented in Table 3. The overall GA was the shortest in acute inflammation and maternal vascular malperfusion, and the longest in chronic inflammation, but the differences were smaller than those suggested by Fig. 1, because the bimodality of GA distribution for some variables was blurring the differences in the averages. The high prevalence of congenital anomalies in this material is striking. This was due to their association with a high percentage of maternal diabetes mellitus and referral of such cases to the children’s hospital. Fetal anomalies (malformations — frequently genetic- or disruptions, extrinsic or intrinsic) were common indications for follow-up and delivery in our children’s hospital, or infants were transferred from other hospitals. In the latter instance, only the cases where the associated placentas were examined at the Cincinnati Children’s Hospital, were included in this analysis. Other classic types of high-risk pregnancy such as hypertensive diseases of pregnancy were less prevalent in this material. Cesarean section deliveries were moderately common, but EXIT procedures [25] were understandably very frequent. The two most common abnormal statistically significant clinical phenotypes/variables found in groups 1–6 were: acute inflammation 5, chronic inflammation 4, MVM 4, FVM, proximal 3, FVM, distal 6, and SPI 0, respectively, thus distinguishing the acute chorioamnionitis and subchorionitis and distal villous fetal vascular malperfusion as the most clinically important patterns of placental injury.

**Table 3:** Clinical phenotypes

Variable	Group 1. Acute inflammation	Group 2. Chronic inflammation	Group 3. Maternal vascular malperfusion	Group 4, Fetal vascular malperfusion, proximal	Group 5. Fetal vascular malperfusion, distal	Shallow placental implantation	F or Chi-square	P
Number of cases	781	412	1012	1401	598	1297		
Clinical outcomes								
Gestational age	*33.1 ± 6.4*	35.3 ± 4.9	*33.3 ± 5.5*	34.9 ± 4.9	34.2 ± 5.3	34.1 ± 5.4	20.235	**0**
Poor or absent prenatal care	31 4.0%	8 1.9%	34 3.4%	41 2.9%	23 3.8%	38 2.9%	5.154	0.392
Multiple pregnancy	50 64%	20 4.8%	60 5.9%	83 5.9%	36 6.0%	90 6.9%	2.903	0.71
Gestational hypertension	45 3.8%	26 6.3%	65. 6.4%	96 6.81%	45 5.8%	70 5.4%	4.44	0.49
Preeclampsia	33 4.2%	*37 9.9%*	54 5.3%	99 7.1*%*	*47 7.9%*	43 3.3%	35.49	**0.0000012**
Chronic hypertension	30 3.8%	19 4.6%	57 5.6%	58 4.1%	21 13.5%	41 3.2%	9.87	0.079
Diabetes mellitus	66 8.4%	52 12.6%	111 11.0%	154 11.0%	82 13.7%	123 9.5%	13.63	0.018
Substance abuse/smoking	93 11.9	42 10.2%	114 11.3%	133 9.5%	65 10.9%	128 9.9%	4.47	0.48
Oligohydramnios	78 10.0%	34 8.2%	103 10.2%	131 9.3%	71 11.9%	123 9.5%	4.68	0.46
Polyhydramnios	43 5.5%	23 5.6%	71 7.0%	*136 9.7%*	*64 10.7%*	95 7.3%	23.73	**0.00024**
Premature rupture of membranes	170 21.8%	43 10.4%	99 9,8%	183 13.1%	70 11.7%	169 13.0-%	66.12	**0**
Antepartum hemorrhage	*100 12.8%*	31 7.5%	*113 11.2%*	105 7.5%	40 6.7%	133 10.2%	27.99	**0.000036**
Meconium-stained amniotic fluid	134 17.2%	60 14.6%	117 11.6%	187 13.3%	34 5.7%	133 10.2%	50.79	**0**
Umbilical cord compromise	102 13.1%	55 13.3%	119 11.8%	185 13.2%	92 15.4%	131 10.15	13.35	0.020
Abnormal fetal heart rate tracing	157 20.1%	104 25.2%	197 19.5%	298 21.3%	125 20.9%	246 19.0%	8.87	0.11
Abnormal umbilical artery dopplers	27 3.5%	*32 7.8%*	*87 8.6%*	81 5.8%	34 5.7%	78 6.0%	22.73	** 0.00038**
Abnormal 3rd stage of labor	80 10.2%	34 8.2%	95 9.4%	144 10.3%	65 10.9%	140 10.8%	5.61	0.35
Delivery methods								
Induction of labor	169 21.6%	95 23.1%	222 21.9%	328 23.4%	161 27.1%	273 21.0%	9.37	0.095
Cesarean section	*289 37.0%*	95 23.1%	222 21.9%	328 23.4%	*162 27.1%*	273 21.0%	80.01	**0**
EXIT procedure	22 2.8%	22 5.3%	60 5.9%	*104 7.4%*	52 8.7%	79 6.1%	26.19	**0.000082**
Fetal/neonatal outcomes								
Neonatal death	102 13.1%	37 9.0%	114 11.3%	140 10.0%	67 11.2%	133 10.2%	7.24	0.20
Non-macerated stillbirth	44 5.6%	16 3.9%	56 5.5%	55 3.9%	30 5.0%	54 4.2%	6.58	0.25
Macerated stillbirth	*150 19.2%*	55 13.3%	184 18.2%	187 13.3%	*122 20.4%*	146 11.3%	49.26	**0**
Fetal growth restriction	103 13.2%	*96 23.3%*	*240 23.7%*	225 16.1%	132 22.1%	244 18.8%	48.44	**0**
Congenital anomalies	190 24.3%	150 36.4%	315 31.1%	*672 48.1%*	*302 50.5%*	503 38.8%	179.09	**0**

Bold font – differences statistically significant after Bonferroni correction; Italics – two most abnormal phenotypes in statistically significant categories

Of placental phenotypes (Table 4), statistical significance was first calculated among the 6 groups, and second, for other individual variables which did not define the 6 studied groups. Placental weight was the lowest in Groups 1 and 3, proportionally to gestational age. Consistent with clinical differences among the groups, acute inflammation, MVM and large vessel (proximal) FVM groups showed no significant statistical differences among the basic groups of placental patterns/lesions analyzed. Only chronic villitis of unknown etiology, distal villous FVM as a group (Group 5), and SPI (Group 6) and its two components (membrane microscopic chorionic cysts and excessive chorionic disc extravillous trophoblasts) showed statistically significant differences. Chronic villitis was most common in Group 1 and 6, distal villous FVM in Group 2 and Group 3, and SPI in groups 3,4 and 5. Of other placental phenotypes not being included in definition of the 6 groups, acute hypoxic lesions (laminar membrane necrosis and meconium macrophages) were least common in the chronic inflammatory pattern group (Group 2), stillbirth-related stem luminal vascular abnormalities were most common in Groups 1, 3 and 5, retroplacental hematomas in Groups 1 and 3, and intervillous thrombi in Groups 3, 4 and 5. Choriodecidual hemosiderosis was most common in the acute inflammation group, while hypercoiled umbilical cord in FVM (proximal and distal). The two most common statistically significant abnormal placental phenotypes were seen in groups 1–6: acute inflammation 6, chronic inflammation 1, MVM 7, FVM, proximal 4, FVM, distal 9, and SPI 0, respectively, thus distinguishing the MVM and distal villous FVM as being the most frequently statistically significantly associated with other placental abnormalities.

**Table 4:** Placental phenotypes

Variable	Group 1 Acute inflammation	Group 2 Chronic inflammation	Group 3. Maternal vascular malperfusion	Group 4. Fetal vascular malperfusion, proximal	Group 5. Fetal vascular malperfusion, distal	Group 6. Shallow placental implantation	F or Chi-square	p
Number of cases	781	412	1012	1401	598	1297		
Placental weight (grams ± standard deviation)	*357.0 ± 167.8*	396.4 ± 196.7	*362.5 ± 205.7*	407.0 ± 193.9	394.9 ± 221.6	384 ± 183.6	9.989	**0**
Acute inflammation (p groups 2-6)								
Acute subchorionitis	N/A	64 15.5%	158 15.6%	180 12.8%	83 13.9%	172 13.3%	5.13	0.27
Acute chorioamnionitis, maternal inflammatory reaction	N/A	47 11.4%	88 8.7%	119 8.5%	57 9.5%	93 7.2%	8.2	0.084
Acute chorioamnionitis, fetal	N/A	36 8.7%	78 7.7%	122 8.7%	40 6.7%	119 9.2%	4.16	0.38
Chronic inflammation (p groups 1, 3–6								
Chronic villitis of unknown etiology	*147 18.8%*	N/A	134 13.2%	203 14.5%	*101 16.9%*	155 11.9%	22.55	**0.00015**
Plasma cell deciduitis	48 6.1%	N/A	66 6.5%	76 5.4%	41 6.9%	61 4.7%	5.59	0.23
Maternal vascular malperfusion (p grouprs 1,2. 4–6)								
Maternal vascular malperfusion	324 41.5%	181 43.9%	N/A	594 42.4%	293 49.0%	574 44.3%	9.49	0.050
Hypertrophic decidual arteriopathy	170 17.9%	98 23.8%	N/A	317 22.6%	157 26.5%	306 23.6%	4.41	0.36
Hyaline necrosis/atherosis	41 7.5%	28 6.8%	N/A	84 6.0%	45 7.5%	81 6.2%	3.37	0.50
Villous infarction, established (> 5% of placental parenchyma)	93 11.9%	50 12.1%	N/A	148 10.6%	91 15.2%	145 11.2%	9.33	0.053
Intravillous hemorrhage	56 7.2%	20 4.8%	N/A	53 3.8%	30 5.0%	58 4.5%	13.4	0.011
Patterns of chronic hypoxic placental injury	122 15.6%	76 18.4%	N/A	272 19.4%	134 22.4%	284 21.9%	15.36	0.0040
Pre-uterine	32 4.1%	15 3.6%	N/A	67 4.8%	25 4.2%	53 4.1%	1.47	0.83
Uterine	63 8.1%	42 10.2%	N/A	137 9.8%	74 12.4%	153 11.8%	10.33	0.035
Post-uterine (diffuse, global)	27 3.5%	19 4.6%	N/A	68 4.8%	35 2.3%	28 2.2%	7.72	0.102
Fetal vascular malperfusion, proximal (p groups 1–3, 6)								
Fetal vascular malperfusion, proximal	421 53.9%	249 60.4%	594 58.7%	N/A	N/A	730 56.3%	6.49	0.090
Fetal vascular ectasia	314 40.2%	159 38.6%	396 39.1%	N/A	N/A	567 43.7%	6.61	0.085
Luminal vascular thrombi	201 25.7%	108 26.2%	264 26.1%	N/A	N/A	315 24.3%	1.29	0.73
Intramural fibrin deposition	88 4.3%	62 15.0%	130 12.8%	N/A	N/A	173 13.3%	3.76	0.29
Stem vessel obliteration	135 17.3%	84 20.4%	178 17.6%	N/A	N/A	282 21.7%	11.11	0.011
Fetal vascular malperfusion, distal (p groups 1–3, 6)								
Fetal vascular malperfusion, distal	242 31.0%	*159 38.6%*	*392 38.7%*	N/A	N/A	429 33.1%	16.19	**0.0010**
Recent, clustered endothelial fragmentation/stromal vascular karyorrhexis	97 12.4%	61 14.8	134 13.2%	N/A	N/A	190 14.6%	2.65	0.45
Established, clustered avascular/sclerotic villi	194 24.8%	117 28.4%	317 31.3%	N/A	N/A	339 26.1%	11.52	0.0092
Remote, clustered villous mineralization	69 8.8%	26 6.3%	98 9.7%	N/A	N/A	100 7.7%	5.51	0.14
High grade distal villous malperfusion	104 13.3%	65 15.8%	154 15.2%	N/A	N/A	151 11.6%	8.22	0.042
Shallow placental implantation (p groups 1–5)								
Shallow placental implantation	349 (44.7%)	197 (47.8%)	525 (51.9%)	*730 (52.1%)*	*329 (55.0%)*	N/A	18.80	**0.00086**
Membrane chorionic microcysts	58 7.4%	58 14.1%	131 12.9%	*205 14.6%*	86 14.4%	N/A	26.38	**0.000026**
Chorionic disc chorionic microcysts	124 15.9%	70 17.0%	190 18.8%	262 18.7%	111 18.6%	N/A	3.67	0.45
Decidual multinucleate trophoblastic giant cells, clustered	182 23.3%	79 19.2%	282 27.9%	321 22.9%	155 25.9%	N/A	15.64	0.0035
Excessive amount of extravillous trophoblast in chorionic disc	140 17.9%	74 18.0%	*250 24.7%*	309 22.1%	*154 25.7%*	N/A	**20.65**	**0.00037**
Placenta creta (including basal plate myometrial fibers)	96 12.3%	51 12.4%	135 13.3%	191 13.6%	91 15.2%	N/A	2.96	0.56
Other placental phenotypes (p groups 1–6)								
Meconium in membranes	*346 44.3%*	60 14.6%	379 37.4%	559 39.9%	*239 40.0%*	494 38.1%	112.63	**0**
Laminar necrosis of membranes	40 26.9%	116 24.3%	*330 32.6%*	416 29.7%	*183 31.6%*	408 31.5%	227.46	**0**
Luminal vascular abnormalities of stem villi (stillbirth-related)	*106 13.5%*	34 8.2%	124 12.2%	117 8.3%	*78 13.0%*	101 7.8%	34.87	**0.0000016**
Increased extracellular matrix of chorionic villi	111 14.2%	51 12.4%	159 15.7%	167 11.9%	96 16.0%	144 11.1%	17.70	0.0033
Retroplacental hematoma	*63 8.1%*	16 3.9%	*92 9.1%*	52 3.7%	22 3.7%	62 4.8%	50.04	**0**
Intervillous thrombus	196 25.1%	115 27.9%	295 29.1%	*467 33.3%*	*234 39.1%*	360 27.8%	43.91	**0**
Erythroblasts in fetal blood	128 16.4%	71 17.2%	201 19.9%	237 16.9%	116 19.4%	185 14.3%	15.37	0.0089
Massive perivillous fibrinoid deposition (> 30% of placental parenchyma)	47 6.0%	26 6.3%	46 4.5%	48 3.4%	29 4.8%	67 5.2%	10.94	0.052
Chorangiosis	70 9.0%	53 12.9%	115 11.4%	159 11.3%	70 11.7%	141 10.9%	5.37	0.37
Chorangioma/chorangiomatosis	16 2.0%	13 3.1%	34 3.3%	40 2.8%	21 3.5%	33 2.5%	4.28	0.51
Choriodecidual hemosiderosis	*59 7.5%*	23 5.6%	*80 7.9%*	59 4.2%	30 5.0%	74 5.7%	19.35	**0.0016**
Villous edema	49 6.3%	24 5.8%	66 6.5%	81 5.8%	42 7.9%	61 4.7%	5.66	0.34
Perivascular stem edema	57 7.3%	34 8.2%	77 7.6%	138 9.8%	60 10.0%	105 8.1%	7.80	0.17
Amnion nodosum/chorion nodosum	41 5.2%	12 2.9%	51 5.0%	67 4.8%	39 6.5%	64 4.9%	6.97	0.22
Hypercoiled umbilical cord	211 27.0%	111 26.9%	269 26.6%	460 32.8%	207 34.6%	360 27.8%	24.69	0.00016
Hypocoiled umbilical cord	56 7.2%	37 9.0%	77 7.6%	88 6.3%	47 7.9%	97 7.5%	4.36	0.50
Marginal insertion of umbilical cord	41 5.2%	26 6.3%	69 6.8%	86 6.1%	39 6.5%	85 6.5%	2.17	0.82
Velamentous insertion of umbilical cord	16 2.0%	8 1.9%	34 3.3%	49 3.5%	18 3.0%	41 3.2%	5.73	0.33
Single umbilical artery cord	35 4.5%	28 6.8%	61 6.0%	91 6.5%	52 8.7%	67 5.2%	13.37	0.020
Other umbilical cord abnormalities	138 17.7%	70 17.0%	205 20.3%	291 20.8%	141 23.6%	246 19.0%	11.17	0.048
Marginate or vallate placenta	41 5.2%	29 7.0%	80 7.9%	135 9.6%	58 9.7%	99 7.6%	16.16	0.0064
Gross chorionic cyst (s)	6 0.8%	6 1.5%	7 0.7%	22 1.6%	10 1.7%	23 1.7%	8.06	0.15
Succenturiate lobe	10 1.3%	10 2.4%	21 2.1%	36 2.6%	14 2.3%	34 2.6%	4.96	0,42

^a^At least 10% of membrane rolls, ^b^at least 3 pseudocysts per membrane roll, ^c^at least 3 pseudocysts per a section of grossly unremarkable chorionic disc, ^d^ > 5 cell islands/placental septa per chorionic disc section; Bold font: p Bonferroni < 0.002 (chi-square), italics – the two most abnormal phenotypes in a category

There was a good positive correlation of chronic inflammation and large vessel FVM and gestational age, and negative correlation between acute inflammation and large vessel FVM (Table 5).

**Table 5:** Mutual linear correlation (r) of 6 patterns of placental injury and gestational age

	Gestational age	Acute inflammation	Chronic inflammation	Maternal vascular malperfusion	Fetal vascular malperfusion, large vessel	Fetal vascular malperfusion, distal villous	Shallow placental implantation
Gestational age	1.00						
Acute inflammation	− 0.38	1.00					
Chronic inflammation	0.71	− 0.21	1.00				
Maternal vascular malperfusion	− 0.57	0.05	− 0.40	1.00			
Fetal vascular malperfusion, large vessel	0.68	− 0.63	0.32	− 0.26	1.00		
Fetal vascular malperfusion, distal villous	− 0.48	0.11	− 0.43	0.57	− 0.03	1.00	
Shallow placental implantation	− 0.39	0.03	− 0.48	0.51	− 0.17	0.48	1.00

## Discussion

### Clinical context

Our results confirm the frequent co-existence of various clinical conditions and patterns of placental injury but reports of prevalence of such patterns in the material classified based on histological grounds are virtually non-existent. Therefore, the interpretation of our findings must be cautious until our findings are confirmed by other investigators. As they come mostly from our Children’s Hospital, the cases studied here constitute a specific population of high-risk pregnancies dominated by congenital anomalies. The classical high-risk pregnancies (hypertensive disease, inflammatory conditions) are less represented. Diabetic pregnancies and fetal growth restriction are common as they are notorious to be associated with fetal congenital anomalies [26]. The population of placentas was divided into 6 groups based on the most common patterns of placental injury (acute inflammation, chronic inflammation, MVM, FVM (large vessel), FVM (distal), and SPI (Table 1). Therefore, this analysis uses a different approach than most classic analyses which compare placental pathologies in clinical etiopathogenetic groups, hence difficulties in finding appropriate references for comparison with our results.

The most advanced GA at delivery was in the large vessel (global) FVM group, which was due to the UC compromise, proved to be frequently associated with fetal congenital anomalies, polyhydramnios, and SPI [8, 27] (Table 3).

Placental pathology of stillbirths at different GAs varies (acute funisitis in extreme preterm birth, uteroplacental insufficiency with parenchymal infarcts in early preterm pregnancies, and umbilical cord complications and infection in term stillbirth [9]. By Ward dendrograms, macerated third trimester stillbirths have multifactorial etiology more likely than the second trimester stillbirths and the likely stasis-induced FVM secondary to occult UC compromise should be sought [12]. By this analysis, macerated stillbirths were common and associated most frequently with acute inflammation, MVM and distal villous FVM (Table 3), confirming our above previous report. Non-macerated stillbirths were most frequently associated with ascending infection (Group 1), albeit still not statistically significant. By this analysis, it was confirmed that the placental abnormalities were less common in the second trimester, other than the acute chorioamnionitis (Figs. 2 and 3) [2].

Interestingly, in this clinical sample, preeclampsia was more common with chorioamnionitis and FVM than with the features of MVM (Table 3). This may be because it was not the primary reason for hospitalization but was associated with conditions of other great obstetrical syndromes [28–30]. Early-onset cases of fetal smallness clustered with cases of poor uteroplacental perfusion whereas late-onset cases did not, as it was reported previously [31].

Maternal diabetes mellitus cases in this material were most common with distal FVM consistent with our report of equal prevalence of lesions of MVM and FVM in diabetic pregnancies. However, several hypoxic lesions and patterns as well as those of SPI were also seen with increased frequencies in diabetic pregnancies, consistent with the literature findings [26]. With the use of E. cadherin/CD 34 immunostain, FVM is as common as MVM in diabetic pregnancies with high prevalence of fetal congenital malformations. This is possibly due to umbilical cord compression evoked by mass-forming fetal anomalies [26] or by fetal heart failure [5].

It is noteworthy that the highest rate of cesarean sections and meconium-stained amniotic fluid was associated with acute chorioamnionitis (Table 3), as reported previously [15]. Clinical umbilical cord compromise was not statistically significantly more common in any group, although not observed clinically (occult). Clinical conditions without umbilical cord compromise were not associated with the increased rate of FVM [27]. The most common single umbilical cord abnormality in this material was hyper-coiling [32]. The detection of placental and umbilical cord abnormalities may help to identify children at increased risk of cerebral palsy. The associations between placental or umbilical cord abnormalities and the risk of cerebral palsy do not vary with GA at birth or sex of the child [33].

### Placental context

The placental weight was understandably the lowest in Groups 1 and 3, consistent with the shortest average GA in those groups (Table 4). The analysis of placental phenotypes showed that there is a significant overlap between various types of placental injury and lesions in individual cases. It was reported that the placental phenotypes clustered statistically significantly with severe preeclampsia in the second trimester; preterm premature rupture of membranes, placental abruption, and fetal growth restriction in the whole 3rd trimester; and abnormally invasive placenta, thick meconium, maternal diabetes mellitus, and substance abuse in term pregnancies [15]. Placental clinicopathological associations are strongest for the second trimester. The awareness of gestational age dependance of some clinical conditions and placental lesions and patterns of injury may be useful in a meaningful clinicopathological explanation of perinatal outcome in individual cases by comparing the observed clinical and placental phenotypes with the dendrograms and multi-dimensional scaling [15].

The group of proximal FVM was the most numerous, followed by SPI and MVM. This confirms that in such a population of placentas as ours, FVM is the most common type of placental injury, the proximal more common than distal, suggesting the dominant umbilical cord compromise etiology in mass-forming fetal anomalies or fetal heart failure associated with fetal heart anomalies [34]. The inflammatory patterns and distal FVM were less common (Table 1). This is different from unselected pregnancies, where the inflammatory and MVM patterns associated with hypertensive conditions of pregnancy would most likely predominate [4]. Nevertheless, the frequency of clinical umbilical cord compromise did not reach statistical significance in this material (Table 3), but the FVM frequencies were statistically significant, likely because of the occult nature (not diagnosed antepartum) of the umbilical cord compromise.

Fetal vascular ectasia and fetal vascular thrombi showed lower GA at birth (about 1 week) than intramural fibrin deposition and stem vessel obliteration. The distinction between intramural fibrin deposition and nonocclusive mural thrombi is problematic, however [35]. Nevertheless, the individual large proximal FVM lesions do not portend significant additional risk to the fetus. They were usually less frequent with other placental lesions/patterns (Table 4). The coexistence of 3 or 4 lesions was associated with the most advanced gestational age, fetal congenital anomalies, distal villous FVM particularly high grade, chorangioma/chorangiomatosis, hypercoiled umbilical cord, periarterial stem edema and marginate or vallate placenta. The more common large vessel FVM lesions, the higher GA at birth [24]. Large vessel FVM and isolated distal villous recent FVM are seen in more advanced pregnancies than established distal villous and ongoing FVM, also in placentas of liveborn children [34]. Global FVM is associated with umbilical cord pathology and neonatal encephalopathy but overall, the recurrence risk of FVM is low [7].

Some authors postulate that once segmental FVM is recognized, it is not necessary to make an additional diagnosis of global FVM. Segmental FVM has 72 or more hours of duration [11], whereas global FVM is usually of short duration, therefore more common. Because of the longer time needed for its development, distal FVM lasts for a longer period before delivery, hence it may portend poorer prognosis for the fetus than large vessel FVM [23]. It is associated with the shortest GA at delivery, more frequent perinatal mortality, the smallest placental weight, and more common intra-villous hemorrhage, erythroblastosis of fetal blood, hypertrophic decidual arteriopathy, and fetal vascular thrombi [35].

Segmental mineralization has an average GA more advanced than diffuse mineralization, but still in early pre-term GA range (29 weeks) [21]. However, the terminal villus involvement requires longer duration for its development and may be therefore more consequential than the proximal vascular lesions which are of shorter duration [23]. Altogether, high grade FVM irrespective of pattern is associated with neonatal encephalopathy [7] and central nervous system anomalies [36].

The analysis confirmed the value of CD34 immunostaining which serves many diagnostic purposes [37, 38]. When compared with H&E staining, the CD34 increases sensitivity and/or upgrades FVM in placental examination in stillbirths but not livebirths [37, 38], but the most important is the diagnosis of the recent FVM. By highlighting the distal villous endothelial fragmentation, the double E-cadherin/CD34 immunostain can reveal the recent FVM not seen on hematoxylin–eosin–stained sections. We routinely perform the stain on a grossly unremarkable placental section predominantly from pregnancies with mass-forming fetal anomalies and umbilical cord complications. The stain can upgrade the FVM, and/or reveal its temporal heterogeneity, both useful in establishing the cause of fetal death or poor neonatal condition. It also highlights the basement membranes of syncytiotrophoblasts which in conjunction with endothelial staining are helpful in the diagnosis of widening thereof in distal villous hypomaturity/dysmaturity. It can distinguish mineralized stem occluding thrombi from mineralized trophoblastic pseudo inclusions, the first outlined by CD34, the second by E-cadherin, thus helping to differentiate FVM from possible placental aneuploidies. The E-cadherin component helps in the diagnosis of trophoblastic lesions of shallow placental implantation featuring the increased number of extravillous trophoblasts in placental membranes and chorionic disc [38]. The average GA at delivery of recent and late FVM (33–34 weeks) was a month more advanced than remote FVM [22]. Distal FVM lesions are associated with chronic placental hypoxia and fetal growth restriction, justifying its inclusion to the category of lesions related to “Great Obstetrical Syndromes” (preeclampsia, fetal growth restriction, preterm labor, preterm premature rupture of membranes, late spontaneous abortion, and abruptio placentae) [28]. Distal villous involvement is the most consequential both from the clinical and placental point of view as it is the most associated with abnormal clinical and placental phenotypes. Recognizing placental FVM may raise awareness of the increased risk of neonatal systemic thrombotic pathology.

Acute inflammation is associated with abnormal clinical and placental phenotypes but is less common in our placental material of Children’s Hospital. It’s lesions are not common but showed the highest proportion of chronic inflammation and large vessel FVM (Table 4). This is not unusual that infections may produce a component of autoimmunity [2], resulting in the coexistence of acute and chronic inflammation, which we showed in our material (Table 4).

Chronic inflammatory lesions (villitis of unknown etiology, chronic histiocytic intervillositis) were the least common group in this material (Table 4). They were reportedly associated with autoimmune disease (SLE, Sjögren’s and autoimmune thyroid disease, obstetric antiphospholipid syndrome and fetal/neonatal autoimmune thrombocytopenia) [39]. They featured the longest GA at delivery (Table 3), but in preterm placentas they may be clinically asymptomatic [40]. However, quite unexpectedly, chronic villitis of unknown etiology was most frequent in association with acute inflammation, but acute inflammatory lesions did not show statistically significant associations anyway in this material. We confirmed the high prevalence of fetal growth restriction in chronic villitis of unknown etiology [4]. It must be stressed that the adjacent lesions of distal villous fetal vascular malperfusion and chronic villitis must be carefully evaluated not to overdiagnose the fetal vascular malperfusion [7, 38].

In SPI, fewer extravillous trophoblasts invade into the uterus and its spiral arterioles, and more of them remain in the placenta [8]. Most commonly, SPI is seen in both early and late onset preeclampsia, while concomitant acute and chronic hypoxic lesions reflect severity of preeclampsia seen more frequently with early-onset preeclampsia [30]. Occult placenta creta should be recognized as a lesion of shallow placental implantation and abnormal trophoblast invasion rather than decidual deficiency only [41]. Without taking into consideration the occult placenta creta spectrum, SPI (at least one lesion), showed no correlation with GA, with average GA in SPI being same (33 weeks) as in cases without SPI [8]. However, with inclusion of sub-clinical placenta creta, SPI was most common in mid-third trimester (Fig. 2). In this material, of the 6 groups analyzed, SPI showed not only overall correlation with MVM and both types of FVM, but membrane chorionic microcysts were seen most commonly in association with chronic inflammatory lesions, and excessive amount trophoblasts in chorionic disc were seen more commonly with lesions of MVM and FVM. However, two of its lesions, chorionic microcysts and clusters of avascular villi show strong correlation with GA at delivery [18]. SPI is more sensitive but less specific than other abnormal placental phenotypes (Table 4) but it recurs in subsequent pregnancies [42].

Of other placental lesions not being components of the 6 groups, discussed above, IVTs were statistically significantly associated with FVM, both large vessel and distal villous (Table 4). Acute fetal distress (abnormal FHR and clinical and histological meconium) was increasing in frequency with GA and were statistically significantly most common in full-term pregnancies. Acute hypoxic lesions, including meconium macrophages, were most common in acute inflammation, and membrane laminar necrosis in MVM, distal villous FVM and SPI (Table 4).

It was reported previously that the clinical conditions linked to in-utero hypoxia (preeclampsia, diabetes mellitus, fetal growth restriction) and their placental associations (atherosis, membrane chorionic microcysts, chorangiosis) were the dominating etiopathogenetic factor in the mid third trimester (late preterm, and near-term), and acute fetal distress in full-term births [9].

Retroplacental hematomas were more common in acute inflammation and MVM, the patterns associated with placental abruption and clinical antepartum hemorrhage (Table 4). The same groups showed choriodecidual hemosiderosis, a lesion of chronic placental abruption. Hypoxic placental lesions can occur without clinical risk factors but with a tendency to recur in future pregnancies [31].

Hypercoiled umbilical cord was commonly seen in both types of FVM, likely to be associated with occult umbilical cord compromise, as umbilical cord compromise was infrequently diagnosed clinically in this material (Table 3). Stillbirth-related stem luminal vascular abnormalities [43] were prevalent in acute chorioamnionitis and distal villous FVM (Table 4), where the macerated stillbirth was also most prevalent (Table 3).

The limitation of this study is a very selective group of high-risk pregnancies analyzed, with mostly pregnancies complicated by fetal congenital malformations. Also, no true control groups were provided, rendering the diagnosis purely comparative. Also, placentas with stillbirths were analyzed together with those with live births but this approach was used also in the author’s previous publications with good results.

The strength of the study was the analysis of the routinely performed double E-cadherin/CD34 immunostain which increases the sensitivity of placental examination for FVM, but which is not yet widely adopted by placental pathologists. Also, the analysis of several newly adopted placental lesions by the authors was performed.

In summary, most placental lesions and patterns of injury are not isolated (the constellation of findings concept), neither they are strikingly characteristic of a clinical entity. This is consistent with the opinion that placental lesion multiplicity is deciding for the maternal and fetal outcome [44], and that so-called placental overlap lesions and patterns [13, 16] are more consequential for pregnancy than single lesions.

## Data Availability

Data are available at request.
